# Molecular Mechanisms Governing the Stem Cell’s Fate in Brain Cancer: Factors of Stemness and Quiescence

**DOI:** 10.3389/fncel.2018.00388

**Published:** 2018-11-19

**Authors:** Valeriia Gulaia, Vadim Kumeiko, Nikita Shved, Eduardas Cicinskas, Stanislav Rybtsov, Alexey Ruzov, Alexander Kagansky

**Affiliations:** ^1^Centre for Genomic and Regenerative Medicine, School of Biomedicine, Far Eastern Federal University, Vladivostok, Russia; ^2^National Scientific Center of Marine Biology, Far Eastern Branch of Russian Academy of Sciences, Vladivostok, Russia; ^3^Department of Cellular Biology and Genetics, School of Natural Sciences, Far Eastern Federal University, Vladivostok, Russia; ^4^Laboratory of Pharmacology and Bioassays, School of Biomedicine, Far Eastern Federal University, Vladivostok, Russia; ^5^Institute for Stem Cell Research, Medical Research Council Centre for Regenerative Medicine, University of Edinburgh, SCRM Bioquarter, Scotland, United Kingdom; ^6^Wolfson Centre for Stem Cells, Tissue Engineering and Modelling (STEM), Division of Cancer and Stem Cells, School of Medicine, Centre for Biomolecular Sciences, University of Nottingham, University Park, Nottingham, United Kingdom

**Keywords:** cellular quiescence, neural stem cell, cancer stem cell, niche, glioma

## Abstract

Cellular quiescence is a reversible, non-cycling state controlled by epigenetic, transcriptional and niche-associated molecular factors. Quiescence is a condition where molecular signaling pathways maintain the poised cell-cycle state whilst enabling rapid cell cycle re-entry. To achieve therapeutic breakthroughs in oncology it is crucial to decipher these molecular mechanisms employed by the cancerous milieu to control, maintain and gear stem cells towards re-activation. Cancer stem-like cells (CSCs) have been extensively studied in most malignancies, including glioma. Here, the aberrant niche activities skew the quiescence/activation equilibrium, leading to rapid tumor relapse after surgery and/or chemotherapy. Unraveling quiescence mechanisms promises to afford prevention of (often multiple) relapses, a key problem in current glioma treatment. This review article covers the current knowledge regarding normal and aberrant cellular quiescence control whilst also exploring how different molecular mechanisms and properties of the neighboring cells can influence the molecular processes behind glioma stem cell quiescence.

## Factors Regulating Normal Cell Quiescence

Stem cells are classically defined as undifferentiated cells possessing a unique ability to produce differentiated daughter cells whilst their population maintains its stem cell state by self-renewal (Weissman, 2000). Cancer stem-like cells (CSCs), or tumor-initiating cells, are defined as a self-renewing subpopulation of cells within the bulk tumor mass that have the ability to recapitulate the entire cell repertoire of the whole tumor (Chen et al., 2012).

Cell quiescence is characterized as a condition of reversible G0 phase from which cells can escape following appropriate physiological cues. The cell quiescence is not only a dormant state but instead an effectively maintained and directed condition, while several molecular pathways permit the quick re-entry into the cell cycle (Cheung and Rando, 2013). The concept of cell quiescence has changed over time. Previously, it was generally thought that cells are forced to enter the dormant G0 phase following nutrient deprivation or contact inhibition, in order to avoid terminal differentiation or senescence, as this condition was considered irreversible. Presently, it is largely accepted that cells, particularly stem cells, enter a quiescent state to preserve their self-renewing capacity and avoid genetic perturbations caused by frequent division. Currently, much attention is centered around the dynamic control of the quiescent state of normal stem cells, which enables them to retain key properties and genomic stability over a macro-organism lifetime. A subset of tissue-specific stem cells is maintained in a dormant state over prolonged time, ensuring tissue replenishment and regeneration in normal and pathological conditions. Due to a particular metabolic and gene expression state, quiescent stem cells can be distinguished by their low RNA content (Fukada et al., 2007) and the absence of proliferation markers (Gerdes et al., 2014), as well as by label retention, explicitly demonstrating their low turnover. However, it was recently shown that using label retention assays alone is insufficient to properly identify quiescent stem cells (Li and Clevers, 2010).

Thus, cell quiescence is regulated by both intrinsic and extrinsic mechanisms. These include various transcription factors, checkpoint regulators and protein kinases/phosphatases, as well as extrinsic regulators, such as cell-to-cell interactions, components of extracellular matrix (ECM) and etc. Both types of regulators are thought to be equally important in facilitating the dormant stem cell state and preventing their exhaustion.

### Intrinsic Regulating Factors

Signaling molecules participating in the regulation of stem cell quiescence include the tumor suppressors p53 and retinoblastoma protein (RB), cyclin-dependent protein kinase (CDK) inhibitors (specifically, p21, p27, p57) and the Notch pathway (Cheung and Rando, 2013). As expected from a non-proliferative phenotype, the quiescent stem cell signatures are recognized as the marked downregulation of genes involved in DNA replication and cell cycle progression. Genes that are frequently downregulated in quiescent cells include those encoding the important cell cycle check point regulators, such as cyclin A2 and cyclin E2, as well as survivin which mediates apoptosis evasion. Genes associated with proliferation and mitochondrial function are also downregulated in quiescent cells. Mitochondrial ATP production is a hallmark of stem cell activation, and thus low expression of cytochrome C (*CYCS*), an active participant of electron transport chain, characterizes low metabolic activity of the quiescent stem cell.

Conversely, genes upregulated in these cells include those encoding signaling proteins involved in transcriptional regulation and stem cell fate decisions such as the key Notch pathway regulator—forkhead box O3 (*FOXO3*) and H3K27 histone methyltransferases—enhancer of zeste homologs 1 and 2 (*EZH1*, *EZH2*). For example, *EZH2* knockout impairs muscle stem cell proliferation and activates expression of non-muscle lineage genes. By contrast, *EZH2* overexpression in hematopoietic stem cells (HSCs) prevents this pool from exhaustion (Hidalgo et al., 2012). EZH1 is a part of a noncanonical Polycomb repressive complex-2 (PRC2) mediating H3 methylation with main function of preserving pluripotency in embryonic stem cells. Together, these investigations propose a critical role of epigenetic mechanisms in regulating stem cell quiescence (Shen et al., 2008).

Adult neural stem cells (NSCs) represent a good model for quiescence investigation as almost all NSCs in the brain are quiescent (Fuentealba et al., 2015; Furutachi et al., 2015), so these cells are being extensively studied for deciphering the molecular mechanisms of dormant state. For example, Rho-GTPase Cdc42, which is a non-canonical Wnt (ncWnt) signaling target, was found to sustain the quiescent state of neuronal stem cells. It was suggested that activation of Cdc42 regulates the expression of specific factors responsible for stem cell identity and anchorage to their niche. Glial and neuronal lineages originate from the intermediate transit-amplifying neural progenitors (type C cells), arising from NSCs (type B cells) of the subventricular zone (SVZ; Lim and Alvarez-Buylla, 2014). Moreover according to the recent literature, vascular cell adhesion molecule-1 (VCAM-1) and N-Cadherin are necessary to maintain quiescent NSCs (qNSCs) within the apical niche, while absence of these proteins disrupts quiescence and results in uncontrolled cell activation, proliferation and differentiation, leading to rapid senescence (Kokovay et al., 2012). NSC adhesion to the apical niche is maintained through the ncWnt signaling, which in turn regulates Notch signaling activity. Strikingly, as a result of a demyelination injury, tissue homeostasis and repair depends on the downregulation of the ncWnt/Cdc42 axis and activation of canonical Wnt (cWnt)/β-catenin signaling in SVZ NSCs (Chavali et al., 2018).

### Extrinsic Regulating Factors

Neighboring cells surrounding NSCs are also important. Ependyma, astrocytes, active NSCs and neuroblasts present the Notch ligands Jagged1 and Dll1 that promote NSC self-renewal through Notch signaling (Ernst et al., 2014). The bone morphogenetic protein (Bmp) ligands and receptors are expressed by qNSCs, which together with Notch, Wnt, insulin-like growth factor 2, vascular endothelial growth factor (VEGF), and EGF signaling pathways regulate quiescence, proliferation and differentiation in the adult neurogenic niche (Llorens-Bobadilla et al., 2015). Normal qNSCs are thought to enter into the cell cycle only rarely, generating actively dividing NSCs that contribute to adult neurogenesis before returning to quiescence. The fate of active NSCs is defined by the total number of neighboring NSCs in a shared niche. Ependymal cells can interfere with the differentiation of NSCs in the glial lineage, since they are capable of producing noggin, an inhibitor of BMP cascade. Additionally, they express CXCR4, the receptor for the stromal cell-derived factor-1 (SDF-1) or CXCL12, which expression is induced by proinflammatory cytokines and association with the Sonic Hedgehog (Shh) signaling cascade during brain development. As the NSC niches are frequently arranged in a perivascular zone, the vessel framework effectively controls the neurogenic procedure. In addition, neurogenesis and vessel formation is controlled by similar elements including IGF-1, bFGF, VEGF and TGF-α (Fidoamore et al., 2016).

There is still more questions then answers as to which molecular mechanisms regulate the transition from quiescent to active proliferative state. Wnt target Tnfrsf19/Troy was very recently found to be a mark of both active and qNSCs (Basak et al., 2018). Transition to proliferation may also be mediated by the high mobility group (HMG) proteins—nonhistone chromatin proteins that affect gene expression by increasing the accessibility of DNA in chromatin for its binding by transcription factors (Thomas and Travers, 2001). Indeed, they have been reported to mediate NSC differentiation. For instance, the HMG AThook 2 (HMGA2) protein is highly expressed in the ventricular zone of the embryonic brain, where NSCs are thought to reside (Sanosaka et al., 2008). Moreover, during embryonic stages, HMGA1 and HMGA2 promote neuronal differentiation while inhibiting astrocyte differentiation of NSCs (Ozturk et al., 2014). HMGB1 and HMGB2 were listed as especially over-expressed during the activation of qNSCs in the adult dentate gyrus (Shin et al., 2015). Addittionally, HMGB2 expression is strongly associated with transition from the quiescent to the proliferative state of NSCs (Kimura et al., 2018).

The Notch pathway was shown to be a key regulator of the quiescence-proliferation balance in stem cells. NOTCH1 is predominantly expressed in active NSCs and transit-amplifying progenitors, while NOTCH3 is preferentially expressed in qNSCs. NOTCH3 knockdown in the lateral wall of adult subependymal zone (SEZ) increases NSC division. Furthermore, NOTCH3 deletion reduces the number of activated qNSCs as a result of antimitotic treatment. Importantly, NOTCH3 deletion preferentially reduces specific subtypes of newborn neurons in the olfactory bulb derived from the lateral walls of the SEZ (Kawai et al., 2017). However, the most recent report pointed Notch2, but not Notch1, being indispensable for maintaining the population of qNSCs in the mouse SVZ, as it represses the cell-cycle and the neuronal differentiation genes (Engler et al., 2018).

Thus, the quiescent state is a practical way for normal stem cells to preserve their pool in tissues while retaining genetic stability and proliferative potential. NSCs can exist in two states: the actively dividing progenitor and the quiescent cell. The transition between these two states is peformed by activating certain molecular pathways, such as Wnt, Notch, Shh, while the certain mechanisms still elude the scientific community. The neighboring cells also provides a control for stem cell regulation by keeping them unresponsive to certain non-threshhold factors, but ensuring fast activation of stem cells in case of injury or other massive cell loss.

## Glioma Stem Cell Molecular Profile

Glioma cells are now believed to originate from a neural stem cell of different commitment state (Alcantara Llaguno and Parada, 2016), therefore “glioma stem cells” and NSCs share common phenotypic markers (CD133, CD44, CD15), as well as activated molecular pathways. Nevertheless, glioma stem cells can be distinguished as they carry particular genetic alterations favoring their continuous division without senescence. Here we describe molecular signatures of different glioma types as they have been thoroughly investigated during the last decade thanks to high throughput techniques, such as whole genome and whole exome sequencing.

### Astrocytoma Molecular Signatures

According to WHO classification released in 2016, all gliomas can be classified by key genetic alterations into three types: astrocytoma, oligodendroglioma and glioblastoma (primary and secondary; Louis et al., 2016). The molecular signature of astrocytomas includes *IDH* mutation, *TP53* mutation and functional loss of *ATRX* (Figarella-Branger et al., 2012). The most frequent *IDH* mutation is the substitution of arginine for histidine at codon 132 (R132H) representing 92.7% of all mutations occurring in the *IDH1* gene. In the *IDH2* gene, R172K substitution represents 65% of all *IDH2* mutations followed by R172M (19%), and R172W (16%; Hartmann et al., 2009). Approximately two-thirds of the IDH-mutant gliomas have an intact 1p/19q, and of these 94% have *TP53* loss of function or other mutation and 86% have *ATRX* inactivation, which is involved in chromatin remodeling and DNA methylation (Brat et al., 2015). Nearly all *ATRX*-mutated gliomas also harbor *TP53* mutations and it was discovered that *TP53* mutations occur first and predispose towards the acquisition of *ATRX* loss of function (Cryan et al., 2014).

### Oligodendroglioma Molecular Signatures

Oligodendrogliomas are characterized by the presence of mutations in IDH1/2 alongside 1p/19q codeletion but almost never acquire alterations in TP53 or ATRX (signature of astrocytomas), as well as copy number amplification (signature of glioblastoma; Reuss et al., 2015). A large proportion of IDH1/2-mutant and 1p/19q codeleted tumors also contain inactivation mutations of tumor suppressor genes—CIC and FUBP1 with a frequency of 20%–30% and 46%–83%, respectively (Bettegowda et al., 2011; Wesseling et al., 2015). Additionally, 96% of tumors with IDH1/2 mutations and 1p/19q codeletion harbor mutation in the TERT promoter (TERT-p; C228T or C250T), which is extremely rare (only 4%) in IDH1/2-mutant 1p/19q intact tumors. However, IDH-wt tumors also commonly acquire TERT-p mutations, conferring cells with the ability to extend telomeres and bypass the Hayflick limit (Eckel-Passow et al., 2015). Interestingly, TERT-p mutation in combination with IDH1/2 alterations brings favorable prognosis, while in IDH-wt tumors it is associated with worse outcome, indicating the existence of an interaction between mutations.

The presence of similar mutation signatures in the IDH gene for astrocytomas and oligodendrogliomas highlights the possibility of a common progenitor cell for these two types of tumor, whereas IDH-wt glioblastomas (GBMs) are believed to arise from a different cell of origin. It is hypothesized that acquisition of certain mutations drives a malignant cell to “differentiation”; either to astrocytoma by obtaining TP53 and ATRX mutations or to oligodendroglioma by losing 1p and 19q (Ohgaki and Kleihues, 2013). However, induction of IDH R132H mutation in neural stem cells leads to either predominant differentiation to neurons (Lu et al., 2012) or to differentiation block and increased apoptosis (Rosiak et al., 2016). Similarly, introduction of IDH1 R132H in combination with TP53 and ATRX knockdown leads to differentiation block in NSCs via transcriptional silencing of SOX2 (Modrek et al., 2017). Thus the incorporation of IDH1 mutation solely results in decreased proliferation ability because of p53-mediated cell cycle arrest, which can be bypassed by TP53 loss (Pirozzi et al., 2017; Zhang Y. et al., 2018), while ATRX mutation confers the cells with infinite growth ability by alternative lengthening of telomeres (Amorim et al., 2016).

CIC is a downstream component of receptor kinase (RTK) pathways (RTK–RAS–RAF–MAPK) and blocks transcription through binding to a regulatory region. It is negatively regulated by RTK signaling which blocks the function of CIC through MAPK-mediated phosphorylation and subsequent degradation. FUBP1 mutations may result in MYC activation or ribosome biogenesis. The additional impact of these alterations on the outcome of patients with 1p/19q codeleted glioma is presently unclear (Wesseling et al., 2015).

### Primary Glioblastoma Molecular Signatures

In contrast to oligodendroglioma and astrocytoma, primary IDH1/2-wt GBMs are more genetically heterogeneous and do not have mutations defining the majority of the cells within the tumor, thus they are referred to as IDH-wt. Absence of marker alteration makes it difficult to suggest the cell of origin and to define initiating or driver mutations for primary IDH-wt GBMs. The majority of primary GBMs are IDH-wt gliomas (95%), while low grade gliomas are IDH-wt in only 20%–25% cases (Parsons et al., 2008). The most frequently altered genes in IDH-wt GBMs include CDKN2A (50%); TP53, epidermal growth factor receptor (EGFR) and PTEN (30%–40%); as well as CDK4, NF1 and Rb1 (12%–15%; Ceccarelli et al., 2016). Almost half of GBMs harbor mutation, rearrangement, or amplification of EGFR (Brennan et al., 2013). Approximately 50% of EGFR-amplified tumors acquire the variant III (EGFRvIII) deletion of exons 2–7 that results in constitutive activation of downstream receptor tyrosine kinase signaling (Verhaak et al., 2010; Appin and Brat, 2015). Approximately 15%–18% of primary IDH-wt GBMs carry PDGFRA amplifications while MDM2 and CDK4 amplifications are present in 5%–15% and 14%–18% of the cases, respectively (Aldape et al., 2015). BRAF V600E mutations are rare in GBMs (Aldape et al., 2015; Takahashi et al., 2015), but can be associated with better prognosis for patients (Vuong et al., 2018). Interestingly, BRAF V600E never occurs alongside IDH1/2 mutations and may also define a subgroup of slowly progressing gliomas with better treatment response (Chi et al., 2013). It is clear that like in tumors with IDH1/2 mutations, BRAF V600E can alter cell methylation profiles, however the reports about extent and mechanism of this methylation changes are controversial (Hinoue et al., 2009; Hou et al., 2012; Bond et al., 2018), emphasizing the necessity for further investigation. Large alterations affecting entire chromosomes are also typical for primary GBMs, specifically chromosome 7 gain and chromosome 9p and 10 loss which present in more than half of primary GBMs. Chromosome 7 contains EGFR, the second most altered gene in IDH-wt GBMs. Chromosome 9 contains tumor suppressor genes such as CDKN2B/p15 and CDKN2A/p16 controlling Rb and p53, and chromosome 10 encompasses PTEN, DMBT1 and LGI1 genes regulating cell growth and cycle progression (Crespo et al., 2011). In primary GBMs, IDH mutations are found at a very low frequency (less than 5%). IDH-mutant gliomas diagnosed as primary GBMs are likely progressed astrocytomas that evaded earlier diagnosis (Ohgaki and Kleihues, 2013), as they frequently contain astrocytoma signature mutations—IDH1 (85%), TP53 (81%) and ATRX (71%; Liu et al., 2012). The transition of anaplastic astrocytomas to secondary GBMs is likely to be driven by acquiring chromosome 10q loss, which is characteristic of primary GBMs, but is found in more than 60% of secondary GBMs (Ohgaki and Kleihues, 2013).

Thus, it is likely that oligodendroglioma and astrocytoma arise from a common precursor cell, such as NSC, because they harbor the same mutation in IDH1/2, which is probably the initiating event in gliomagenesis. Primary IDH-wt GBMs possess markedly different key genetic alterations and in general are more heterogeneous. Therefore, it is likely that IDH-wt GBMs arise from a different precursor cell than IDH-mutant gliomas.

### Glioma Stem Cells Molecular Signatures

It is important to note that, to date, it has not been possible to discriminate between the more quiescent, long-term self-renewing NSCs and the more rapidly dividing progenitor cells. In general, CSCs derived from GBM tumors are characterized by similar mutational arrangement. Specifically, typical GBM CSC populations harbor homozygous CDKN2A deletion, EGFR gain (up to four copies), and alterations in KCN5, PLCB2, GDF5 and/or TRMT5. Loss of wild-type TP53 gene occurrs following EGFR amplification of more than four copies, while heterozygous TP53 and PALB2 mutations occurr upon EGFR amplification beyond this (Piccirillo et al., 2015). Interestingly, certain point *TP53* mutations (c.565G >A and c.451C >T) were shown to mediate therapeutic resistance, as they associat with the relapsing GBM CSC phenotype (Orzan et al., 2017). Additionally, several novel mutations were reported to be hallmarks of GBM CSCs, including alterations in *CENPF* playing a role in chromosome segregation during mitosis, *AJAP1* being tumor suppressor gene, and hypermethylation of the tumor-suppressor gene *EMP3* (Ernst et al., 2009). Several researchers characterized glioma CSCs as a cell population with impaired gene expression rather than certain genomic alterations, for instance, hyperactivity of the cell cycle genes (*IGFBP5, VEGFA, SLC2A3, LGALS3, FAM115C, MT1X, UBC, C4orf3, FAM162A, PPP1R15A, EEF1A1, FTL*; Patel et al., 2014), increased expression of neurodevelopmental TFs (*POU3F2, SOX2, SOX4, SOX11, SALL2* and *OLIG2*; Tirosh et al., 2016), or NSC-related genes (*NFIB, ASCL1, CHD7, CD24, BOC* and *TCF4*; Suvà et al., 2013) were proposed to confer stem-like properties to malignant cells enabling them to convert to CSCs. Additionally, primary GBM cell lines grown as neurospheres were shown to upregulate genes involved in immune modulation—*TNFSF18, CXCL16*, *CX3CL1*, regulation of apotosis—*PRKG1*, growth and survival—*PDGFRA, met, DLL1* compared to the same cells grown as adherent culture (Wilson et al., 2016). While CSCs are being extensively studied in GBM, the presence of this population in low grade gliomas is still arguable, with only a few studies reported to describe genomic and transcriptomic features of CSCs from grade II and III astrocytomas or oligodendrogliomas where they were defined as cells expressing corresponding lineage specific genes along with the elevated expression of cell cycle genes or neural TFs (Tirosh et al., 2016; Venteicher et al., 2017). Meanwhile, evidence suggests that CSCs arise from a common progenitor with signature genetic mutations in *CDKN2A/B*, *EGFR* and *TP53* (Sottoriva et al., 2013).

The molecular signatures of gliomas allow clinicians to make reliable diagnosis and prognosis for patients. How these genetic events drive malignant transformation are being extensively studied but key questions remain. Why do several mutations lead to favorable prognosis and gliomas with less malignant phenotype? What is the mutual effect of mutations on each other?

## Factors Regulating Glioma Stem Cell Quiescence

Little research has been reported to address potential roles quiescence might play in CSC biology. In cancer biology, tumor dormancy designates a frequent clinical phenomenon in which disseminated tumor cells are maintained in a nonproliferating, quiescent state for long time intervals. This phenomenon may occur at early stages of the disease or following therapeutic intervention. Awakening of these dormant cells leads to tumor progression and relapse which may occur after very long periods (Sosa et al., 2014).

The considerable lack of studies specifically investigating the quiescent CSCs is most likely because of difficulties in isolating CSC populations and demonstrating their stem cell properties. Considering that CSCs originate from adult stem cells, the molecular signatures of quiescent CSCs should include such markers as Hes1 (Notch signaling), p21, p16INK4a, Rb, Bmi-1. In fact, quiescent CSCs have similar gene expression signatures to normal dormant stem cells, for example, they downregulate the expression of genes associated with cell cycle, such as, cyclin B1, Cdc20, Cul-1, ubiquitinating cell cycle proteins, and Myc, while upregulate the expression of classical cell cycle controllers, such as, TP53 and MAX-interacting protein 1. Interestingly, quiescent CSC signatures include upregulation of cyclin D2 which is required for G1/S transition in the cell cycle and should normally lead to the evasion of G0 phase. A possible explanation can be that several genes highly expressed in quiescent CSCs ensure their poised state to quickly re-enter the cell cycle if needed. Other signatures of quiescent CSCs include fundamental modulators of key molecular pathways including the Wnt (FZD2 and TCF7L2), BMP (SMAD1) and Notch pathways (Hes1). Interestingly, CSCs acquiring mutations in Rb or its related protein p107 are unable to maintain a quiescent state while still preserving the ability to enter G0 phase (Moore and Lyle, 2011). Here we describe the major pathways that were reported to play a role in cellular quiescence: Wnt cascade, Notch, TP53, Akt/mTOR and hypoxia-inducible factor 1 alpha (HIF1α).

### Canonical/Non-canonical Wnt Cascade

ncWnt signaling has been shown to be a crucial regulator of qNSCs within their niche, through the activation of Rho-GTPase Cdc42 which controls the apical adhesion of qNSCs. Moreover, it was found that transient activation of canonical the Wnt cascade is important for activation and propagation of qNSCs, highlighting the importance of a canonical-non-canonical switch in regulation of temporal quiescence in normal NSCs (Chavali et al., 2018). Additionally, ncWnt maintains quiescent HSCs through key components mediating cell polarization—Flamingo (Fmi) and Frizzled (Fzd) 8 (Sugimura et al., 2012). CD44, CD133 and LGR5 are characteristic cell surface markers of glioma CSCs (Lathia et al., 2015), which are either receptors for cWnt pathway (LGR5), or function as positive regulators of Wnt receptors (CD44; Schmitt et al., 2015), or E-cadherin (CD133; Brossa et al., 2018). In CSCs, cWnt signaling is mostly activated by ligands such as WNT2B and WNT3 which are secreted by tumor niche cells. These genes also can be upregulated as a result of genetic alterations in the cWnt/β-catenin cascade molecules, such as gain-of-function (GOF) mutations in the CTNNB1 (β-catenin) gene and loss-of-function mutations in the signaling regulators, such as APC, AXIN1, AXIN2, RNF43 and ZNRF3 genes (Katoh, 2017). However, cWnt signaling is involved mostly in CSC expansion and tumor growth, but not in preservation of CSC stemness through maintaining a quiescent state. Additionally, it was shown that major cWnt signaling molecules, such as β-catenin and GSK3β are not expressed in glioma cells highlighting the possibility of other pathways driving glioma progression (Zhang H. et al., 2018).

ncWnt signaling through RTKs can activate the PI3K-Akt signaling cascade and affect cell cycle progression. As ncWnt signaling maintains quiescence of stem cells and inhibits cWnt signaling, it has been considered primarily a tumor suppressor. However, experimental data on the ncWnt cascade in glioma is limited and focused mainly on cell invasiveness and migration. To this end, Cdc42 was shown to drive cell mobility in glioma (Okura et al., 2016), while expression of Wnt5a (initiating ligand of ncWnt) in glioma CSCs leads to their differentiation into endothelial-like cells (Hu et al., 2016). Inhibition of Wnt signaling in glioma CSCs leads to loss of stem cell properties (Bhuvanalakshmi et al., 2015) while the expression of Fzd4 in glioma cells is associated with increased stemness and invasiveness (Jin et al., 2011).

It is apparent that the ncWnt cascade plays a significant role in glioma CSC quiescence. It is of interest to explore the role of Rho-GTPases in regulating cell polarity and Fzd receptors for their potential to induce dormancy and highly therapy-resistant glioma cells.

### TP53/Rb

Limited data exists on the extent of which p53 plays a role in quiescence, but there is a lot of evidence of mutant p53 contribution to cancer progression in general and very few studies highlighting its role in CSC maintenance (Chavali et al., 2018), and even less in maintaining quiescence. For example, it was demonstrated that an additional copy of wild type p53 increased quiescence and ciliated cell differentiation (McConnell et al., 2016), by means of recruiting histone deacetylase 1 (HDAC1) to the CD133 promoter which causes reduced histone H3 acetylation and subsequently leads to restricted growth and proliferation (Park et al., 2015). However, mutant p53, especially hotspot GOF mutations were shown to have a driver role in oncogenesis (Olivos and Mayo, 2016). Three most common hotspot mutations found in p53, namely R175, R248Q and R273H, were mapped to the DNA binding domain where they cause single amino acid substitutions (Lawrence et al., 2014). GOF mutant R175H and R273H p53 proteins trigger cell transformation through the involvement of mTOR signaling (Dando et al., 2016). In this case, the mutant p53 mediates signaling through growth factor receptors (GFRs), such as EGFR and hepatocyte growth factor (HGF) receptor, thus promoting PI3K/Akt signaling and downstream activation of mTORC1 (Yallowitz et al., 2015). p53 R175H has been shown to upregulate TWIST1 expression and subsequently decrease the histone H3 methylation upstream of the TWIST1 promoter (Kogan-Sakin et al., 2011). TWIST1 ectopic expression in neural crest cells was implicated in cell lineage determination and differentiation, as it represses pro-neural factors to prevent loss of stem cell properties (Vincentz et al., 2013). Additionally, all three p53 GOF mutations can alter chromatin binding states by upregulating chromatin regulatory genes, including the methyltransferases KMT2A (MLL1) and KMT2D (MLL2), and acetyltransferase KAT6A (MOZ or MYST3), resulting in genome-wide increases of histone methylation and acetylation (Zhu et al., 2015). All these chromatin modulators have been shown to participate in quiescence maintained in healthy stem cells. For example, MLL1 deficiency was shown to abrogate a pool of adult quiescent HSCs (Jones et al., 2015), KMT2D was reported to be essential for maintaining a pool of cardiac precursors (Ang et al., 2016), as well as for preserving the population of adult HSCs and progenitors, because HSCs lacking Mll exhibit increased cell cycling which results in the depletion of quiescent HSCs (Jude et al., 2007). In the same manner, loss of acetyltransferase KAT6A in adult mice leads to the rapid loss of adult HSCs with a concurrent reduction in the population of quiescent cells in G0 phase (Sheikh et al., 2016). Thus, p53 GOF mutations can be of major importance in maintaining quiescent CSCs as they are implicated in gene expression via recruiting transcription factor, binding to promoter sites of protooncogenes, or through regulation of chromatin modifying enzymes, but the molecular mechanism of this regulation in CSCs is still to be discovered.

### HIF1/Notch/p38

Another important contributor to inducing quiescence is hypoxia and its key molecular regulator, HIF1α. HIF1 positive quiescent glioma stem cells were localized in perinecrotic niches within glioblastoma tissue. HIF1α importance in glioma is highlighted by the fact that mutations in IDH1/2 can lead to accumulation of HIF1α (Zhao et al., 2009; Xu et al., 2011), however the most recent report states otherwise (Koivunen et al., 2012). Establishing tumor cell lines from IDH-mt low grade gliomas is challenging as the IDH mutant clones tend to be eliminated during conventional culturing, leading to the conclusion that IDH mutant cells do not possess self-renewing properties (Kelly et al., 2010; Stoczynska-Fidelus et al., 2014). However, it is possible that IDH-mt cells contain HIF1α-induced quiescent stem cells which in normoxic conditions lose their stem potential because of HIF1α inhibition. Additionally, HIF1α and HIF2α were shown to be responsible for acquiring stem cell properties in differentiated glioma cells. Long-term exposure of glioma cell lines containing small stem cell populations to therapeutic stress (temozolomide) conferred differentiated glioma cells with stemness and pluripotency markers, such as CD133, SOX2, Oct4 and Nestin, whilst also upregulating HIF1/2α expression (Auffinger et al., 2014; Lee et al., 2016). However, these two reports demonstrated that HIF1α was coexpressed with proliferation marker ki67, which is against widely accepted idea that HIF overexpression leads to cell cycle arrest through derepression of p21 and p27 (Gardner et al., 2001; Goda et al., 2003). In contrast, HIF2α was shown to have opposite properties and promote cancer cell proliferation through augmentation of c-Myc activity (Gordan et al., 2007). This highlights the problem of lack of comprehensive study in this field, as well as the necessity of taking into account signature mutational profile of the glioma cells to provide more complete information.

HIF1α was also investigated in conjunction with Notch signaling, where it was shown to contribute to glioma CSC maintenance through stabilization of the intracellular domain of Notch (NICD; Qiang et al., 2012). In the brain, Notch signaling controls the state of normal stem cells by facilitating the quiescent state of radial glial cells and preventing neuronal differentiation of progenitors and NSCs (Ables et al., 2011). However, in cancer Notch acts an oncogenic signaling factor, facilitating glioma CSCs transition to quiescent state. Liau et al showed that glioma CSCs can temporarily transition to a dormant state in response to RTK inhibitors. Culturing with high level of RTK inhibitors also led to upregulated expression of various Notch pathway genes which were shown to be essential for cell survival under RTK inhibitory conditions. This condition was shown to be reversible even after prolonged cell cultivation in the slow-cycling state with the capability of restoring high proliferation rate and subsequent downregulation of Notch genes (Liau et al., 2017). However, not all reports refer to Notch signaling as oncogenic driver, Giachino et al. (2015) reported that suppression of key Notch mediators and receptors, such as RBP-Jk, (a transcriptional repressor in the absence Notch molecules), as well as Notch1 and Notch2 induced glioma growth in mice. They proposed a model in which Notch and wild type TP53 act in cooperation and promote tumor growth restriction and cell cycle arrest in glioma cells (Giachino et al., 2015). However, it is possible that these pathways can interplay to promote not only growth restriction but a quiescent state, especially if the Notch cascade coordinates with TP53 GOF mutations.

p38 is a subgroup of mitogen-activated kinases that plays a significant role in cell cycle, differentiation and apoptosis. Inhibition of p38 leads to a decrease in both *in vitro* and *in vivo* glioma stem cell proliferation. The p38 pathway was suggested to affect survival, cell cycle state and differentiation status of glioma CSCs via regulating EGFR trafficking. These observations led to the conclusion that while p38 inhibition of glioma CSCs resulted in diminished proliferative activity, differentiated cancer cells composing the tumor body maintained the slow-cycling glioma CSC state by blocking differentiation into terminal lineages (Soeda et al., 2017).

### PTEN/Akt/mTOR

Numerous investigations have demonstrated that the role of the tumor silencer protein PTEN in cell cycle progression is essential for tumor elements. In low grade gliomas PTEN keeps cells in G1 while the loss of its action is habitually seen in high grade gliomas. PTEN regulates cell cycle related proteins through control of Akt phosphorylation which leads to FoxO1 translocation to the cytoplasm and subsequent repression of Notch pathway (Yue et al., 2017). Additionally, Akt regulates important cell cycle regulators such as E2F2, cdc25a, Cyclin G2 and RBL2 (Choi et al., 2017). The significance of Akt signaling in maintaining quiescent glioma CSCs was also emphasized in previous investigations. The Akt-mTORC2 axis was revealed as a signaling pathway ensuring CSC ability to quit G0 phase. In this case, altered metabolic state of activated cells leads to Akt phosphorylation at T308 by phosphoinositide-dependent kinase-1 (PDK1). Activated Akt in turn phosphorylates mTORC2 component Sin1 leading to a second phosphorylation of Akt at S473 and its full activation (Yang et al., 2015). Moreover, the IKK complex, controlling NF-κB activation and proinflammatory response, can also control mTORC1 and mTORC2 via Akt-dependent regulation (Dan et al., 2016). Additionally, AKT1-low cells were reported to enter a temporarily quiescent state meaning that low Akt levels ensure the ability to induce a dormant state. Quiescent CSCs can likewise upregulate the JARID1B histone demethylase and thereby become more quickly cycling cells with AKT1-high/JARID1B-high profile (Facompre et al., 2016). This represents a relative simplicity of G0 phase exit for CSCs and highlights the importance of epigenetic regulators in this transition, which still lacks proper investigation. Surprisingly, a mutant AKT1 form (E17K substitution) which is considered an oncoprotein, can specifically downregulate the ability of proliferative cancer cells to enter the G0 dormancy state, thus precluding the formation of quiescent CSC pool (Alves et al., 2018). This discovery leads us to the point that some cancer-associated mutations could be advantageous and should be investigated from a different point of view.

The dormant state might be effectively prompted by particular kinases including double specificity tyrosine phosphorylation-managed kinase 1A and B (DYRK1A and DYRK1B). DYRK1A was shown to be crucial for quiescence maintenance by inducing Cyclin D3 degradation, which ensures E2F mediated gene transcription (Thompson et al., 2015). Similarly, the DYRK1B kinase induces Cyclin D degradation by stabilizing the cdk inhibitor p27 and DREAM complex, regulating cell-cycle dependent gene expression (Becker, 2017).

**Figure 1:**
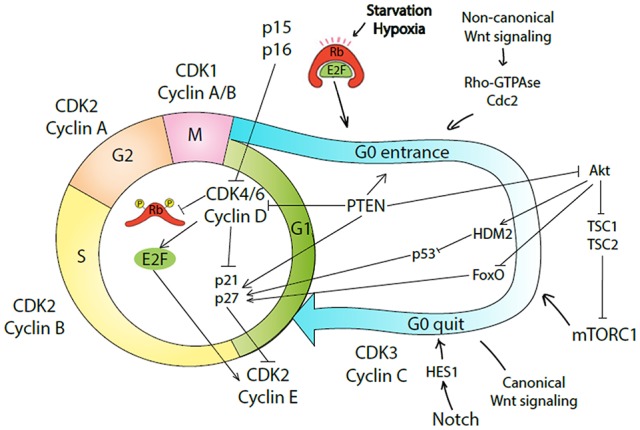
Molecular mechanisms underlying entrance and quite G0-phase of cell cycle by cancer stem-like cells (CSCs). Conventional four phases of the cell cycle are regulated by a specific complex of cyclin and cyclin-dependent kinase (CDK). The regulatory machinery of G1 progression overlaps with decision mechanism about the commitment to quiescence or proliferation. In case of GFs/mitogens availability, CDK4/6/cyclin D complex phosphorylates Rb causing E2F release, which in turn activates cyclin E. CDK2/cyclin E complex drives G1 to S phase transition. CDK4/6/cyclin D complex also removes p21 and p27 inhibition on CDK2. Cells in G1 phase can exit the cell cycle and enter G0 phase as quiescent, senescent, or differentiated cells. In case of nutrition withdraw or hypoxia, Rb renders E2F bound and as a result CDK2/cyclin E complex is inhibited by p21/p27. Alternatively, activation of non-canonical Wnt (ncWnt) signaling causes quiescence induction via Rho-GTPase/Cdc42 complex. PTEN causes G0 entrance by inhibiting CDK4/6/cyclin D complex or Akt pathway. As a result, cells are driven into quiescence. CDK3/cyclin C complex, Notch, canonical Wnt (cWnt) signaling, or Akt/mTORC1 pathway can drive cells to enter cell cycle again. In response to mTORC1 signaling, quiescent cells can transit into the GAlert phase and become poised for rapid cell cycle entry.

Thus, quiescence of glioma stem cells can be regulated by pRb, p53, ncWnt, PTEN and FoxO to enter the quiescent state, and by Akt, mTORC1, cWnt, Notch, CDK3/cyclin C to exit G0 (summarized in Figure 1). The quiescent state of CSCs enables these cells to acquire resistance to commonly used antiproliferative agents. A treatment strategy forcing quiescent CSCs to enter cell cycle was termed the “locked out” strategy, which could bring a therapeutic benefit with acceptable risks to patients with high grade cancers (Takeishi et al., 2013). However, the “locked out” approach may be of too high risk as massive induction of cancer cell proliferation can result in enhanced genetic heterogeneity and selection of highly resistant cell clones (Sosa et al., 2014).

## Symmetric and Asymmetric Stem Cell Division and Maintenance of Glioma Plasticity

Previously it was generally accepted that new neurons can be generated only in the neurogenic phase during embryonic development. However, current studies have clearly demonstrated that neurogenesis is sustained in postnatal stages along with the production of glial cells. The differentiation hierarchy begins from stem cells generating transit amplifying cells, which in turn develop lineage-committed progenitor cells finally resulting in mature cells of various types. The brain analog of embryonic stem cells is derived from the neural tube from which neuroepithelial cells arise. Neuroepithelial cells serve as progenitors for radial glial cells, which then mostly reside in the SVZ surrounding the lateral ventricles and in the subgranular zone (SGZ) of the dentate gyrus within the hippocampus, where these cells persist throughout adulthood. In this context radial glial cells can be termed as NSCs, as they function as a primary progenitor for neurons and astrocytes (Kriegstein and Alvarez-Buylla, 2009).

### Models of Stem Cell Division

There are three primary models describing the nature of neuro- and glio-genesis in the adult human brain: classical (division asymmetry), stochastic (population asymmetry) and disposable stem cell models. The first paradigm proposes that NSCs undergo asymmetric cell division resulting in the emergence two different cells, where only one daughter cell preserves the stem self-renewing properties while the other differentiates into a transient amplifying cell through a series of terminal, symmetric divisions (Bond et al., 2015). In this model, following entry of qNSCs into the cycle, only one daughter cell returns to quiescence, while the other differentiates directly or through a series of terminal divisions. Alternatively, the fate behavior of NSCs could be stochastic in the sense that, upon activation, one active NSC returns to quiescence for every quiescent NSC entering the cell cycle. In the first model, only qNSCs maintain long-term self-renewal potential while, in the second, self-renewal potential is shared by quiescent and active NSCs and achieved only at the population level (population asymmetry model). The third possibility is that the quiescent NSC pool is disposable, in the sense that, once activated, they either differentiate or remain in the cell cycle and become exhausted over time (Basak et al., 2018).

Now there is a new emerging niche restricted model, which suggests that a constant rate of qNSCs become activated sporadically and enter into cycle, while another population of activated NSCs stochastically return to quiescence. During their active phase, NSCs may independently and stochastically choose between cell duplication, giving rise to two active NSCs, and a symmetric differentiating division generating two transit amplifying cells. The division of active NSCs results in cell duplication or differentiation with a probability that depends on the total number of existing NSCs (active or quiescent) in the local niche (Basak et al., 2018).

Recently, growing evidence supporting the disposable model has been provided by genetic lineage tracing and live imaging studies, which suggest that activated NSCs inevitably lose neurogenic potential (Calzolari et al., 2015). Studies utilizing direct visualization of radial glia as well as invertebrate models placed their emphasis on invariant asymmetric cell division (Paridaen and Huttner, 2014). However, despite the fact that evidence supporting either niche-directed asymmetric NSC division or the spontaneous segregation of fate determinants is currently lacking, the asymmetry model is still considered the most widespread in NSC differentiation.

### Pathways Regulating Asymmetric Stem Cell Division

Asymmetric cell division implies the unequal distribution of cell fate determinants between daughter cells which is achieved by the establishment of cellular polarity prior to mitosis (Lewis and Petritsch, 2013). The maintenance of neuroepithelial and radial glial cell polarity is accomplished by tight junctions at the apical and basal cell sides by the adaptor proteins Numb and Numbl, which promote cell cycle withdrawal by downregulating signaling of GFR ERBB2 (Hirai et al., 2017).

**Figure 2:**
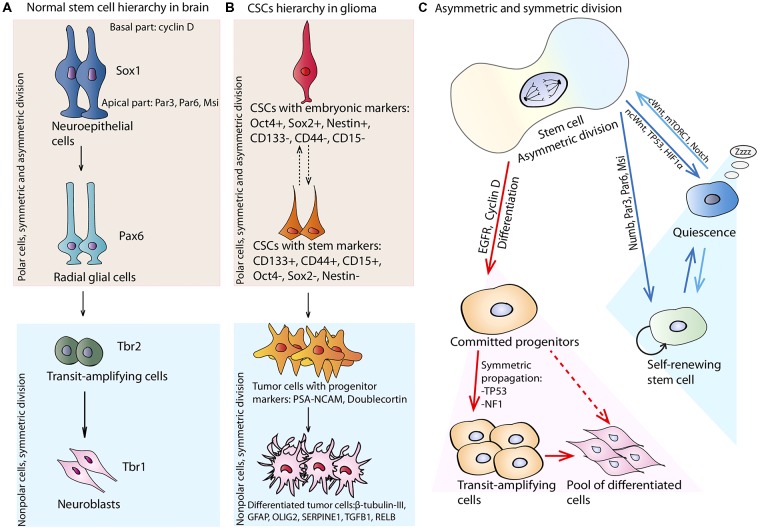
Stem cell hierarchy and division. **(A)** Normal neural stem cells (NSCs) hierarchy: neuroepithelial cells expressing signature transcription factor—Sox1, are polar cells capable of asymmetric division. Apical parts of the cells are enriched with Par3, Par6 and musashi-1 (Msi); basal parts collect cyclin D. Neuroepithelial cell through division can generate more differentiated cells—radial glial cells expressing paired type homeobox 6 (Pax6), which are also polar and can divide asymmetrically. They can produce more differentiated cells—transit-amplifying cells, expressing Trb2 and capable of fast expansion. They can divide only symmetrically and committed to differentiation in neuroblasts. Neuroblasts are symmetrically dividing nonpolar cells expressing Trb1. **(B)** CSCs hierarchy in glioma. Initiating glioma CSCs expressing embryonic transcription factors—Oct4, Sox2 and Nestin. These cells are probably capable of asymmetric division with generation of more differentiated cells expressing stem cell markers—CD133, CD44 and CD15. Separation of glioma CSCs on less differentiated and more differentiated is not strict as the phenotype of these cells highly depends on the phenotype of the cell of origin. CSCs can generate more differentiated cells with progenitor markers: PSA-NCAM and Doublecortin which amplify the general tumor cell pool and can become more differentiated with generation of cells expressing β-tubulin-III, glial fibrillary acidic protein (GFAP), OLIG2, SERPINE1, TGFB1, RELB. **(C)** Stem cell symmetric and asymmetric division. NSC can divide asymmetrically by generating two daughter cells with different fate: one cell inherits EGFR and Cyclin D that poise the cell to rapid symmetric cycling, and the other gets Numb, Par6, Par3 and Msi preventing differentiation programs. If during symmetric or asymmetric division NSC experiences the influence of ncWnt, TP53, or hypoxia-inducible factor 1 alpha (HIF1α) pathways it can enter the quiescent state. If quiescent NSC receives signals from cWnt, mTORC1, or Notch pathways it can enter cell cycle and divide symmetrically or asymmetrically.

According to the division asymmetry model, neuroepithelial cells can simultaneously produce several more differentiated descendants, specifically, radial glial cells, basal progenitors and neurons. Cell fate decisions depend vastly on the predominant TFs and activated molecular pathways inherited by either daughter cell. The formation of radial glial cells occurs when the expression of Sox1 is substituted by the expression of paired type homeobox 6 (Pax6), a pivotal TF for brain patterning (Curto et al., 2014). The production of transient amplifying cells from asymmetrically dividing radial glial cells is determined by reduction of Pax6 expression in favor of Tbr2, a transcription factor expressed in postmitotic basal cells and projection neurons, which is in turn replaced by Tbr1, when cells acquire a terminal neural phenotype (Englund et al., 2005; Ghosh et al., 2017). Proposed hierarchy of NSCs is depicted in Figure 2A. More recently, radial glial cells that leave the ventricle zone to migrate to SVZ and retain basal fiber and Pax6 expression, have been termed as outer glial cells. These cells are capable of asymmetric division with simultaneous production of a basal progenitor and neuron (Wang et al., 2011). The ultimate fate of outer glial cells is regulated by integrin signaling and its differentiation leads to loss of both apical and basal polarity (Fietz et al., 2010).

Both neuroepithelial and radial glial cells are polarized epithelial cells, with different surface regulators expressed on each side, the so-called apical, basal and lateral membranes, each of which can be exclusively characterized by specific molecular markers, such as prominin-1 and cadherins, respectively. When the asymmetric division occurs the more basal daughter cell inherits a positive regulator of G2 progression—cyclin D2, which is concentrated in the basal process of the polar cell, thus predetermining cellular fate (Tsunekawa et al., 2012). Differential cell fate determination is a result of distinct distribution of signaling effectors, including EGFR (Sun et al., 2005), polarity regulators Par3 and Par6 (Gómez-López et al., 2014), and neural RNA-binding protein Musashi-1 (Msi1), all of which were shown to preferentially colocalize apically in the less differentiated daughter cell during asymmetric cell division. Par3 exerts its effect by direct interaction with Numb and Numb-like (Numbl) and thereby modulating its antagonistic effect on Notch signaling (Bultje et al., 2009). Meanwhile, Msi1 augments Notch signaling through the translational repression of Numb, thereby contributing to NSC self-renewal (Okano et al., 2005).

In fact, gliogenesis is a predominant process over neurogenesis in the adult brain. This neurogenic-gliogenic switch, which is a characteristic feature of the developed nervous system, requires the expression of the mitogen activated protein (MAP) kinase kinases Mek1 and Mek2, Notch pathway proteins and the Signal transducer and activator of transcription 3 (STAT3) signaling members. Meanwhile, astrocytes emerge from the glial-restricted precursor expressing A2B5, whereas oligodendrocytes develop from oligodendrocyte progenitor cells expressing such signatures as Olig2, NG2 proteoglycan and platelet-derived GFR-alpha (PDGFRA). Nevertheless, in the adult brain there are two remaining sites of neurogenesis: the SVZ and the SGZ. In these regions, a subpopulation of outer radial glial cells, characterized by glial fibrillary acidic protein (GFAP) and Nestin expression, act as NSCs (Pollen et al., 2015). These NSCs from adult brain, are also known as type B cells, can produce transit-amplifying progenitors, also termed type C cells, which will divide a finite number of times until they become differentiated (Doetsch et al., 1997), and oligodendrocyte precursor cells (OPCs), which can generate neuroblasts (type A cells; Doetsch et al., 1999), and oligodendrocytes (Menn et al., 2006), respectively.

### Glioma CSC Stemness Preservation and Plasticity

Glioma CSCs are thought to arise from NSCs in SVZ. A recent report by Lee et al added a robust confirmation to this theory, as they were able to demonstrate that astrocyte-like NSCs from the tumor free SVZ frequently share similar genetic alterations with matched tumor from the same patient, especially in the TERT promoter region. Although it is possible that early stage glioma cells migrate to the tumor-free SVZ and initiate a pool of NSCs with low level of GBM mutations, it is more likely that astrocyte-like NSCs from the SVZ are precursor cells for glioma CSCs (Lee et al., 2018). As glioma CSCs and NSCs share a lot of common features, glioma CSCs were suggested to have reminiscent differentiation hierarchy characterized by embryonic stem cells at the beginning, which subsequently generate transit amplifying cells, lineage-committed progenitors and mature cells. In order to create a hierarchical model of glioblastoma CSC differentiation, Bradshaw et al. (2016) systematized all the known putative phenotypic markers of GBM CSCs. They divided the GBM CSCs into two classes; those expressing markers associated with an embryonic stem cell phenotype and expressing Nanog, Sall4, Oct-4, Klf4, Sox2, and those that acquired a neuronal stem cell phenotype with CD133 and CD44 expression. There is a possibility to expand this classification by assuming that glioma CSCs can acquire differentiation markers common for normal glia and neurons. In this way, stages of CSC differentiation can be distinguished by rising progenitor cell markers (PSA-NCAM and Doublecortin; van Strien et al., 2011) and terminal differentiation markers of oligodendrocytes (ASCL1, OLIG2, DLL3), astrocytes (GFAP), neurons (β-tubulin III, SYT1 and SLC12A5), and markers of inflamed astroglial cells (SERPINE1, TGFB1, RELB; Sullivan et al., 2014). The probable glioma CSCs hierarchy is represented in Figure 2B. However, cell surface phenotypic markers do not always properly reflect the current cell state, and this model is not fully complete as it is missing the markers of finite differentiation. Additionally, the glioma CSC secretome was found to change during distinct differentiation stages and it was demonstrated that quiescent glioma CSCs do not secrete any cytokines, but maturation stimulation causes them to produce different cytokines at different times, beginning with the production of EGF on the 2nd day of differentiation, MCP1 on the 3rd, VEGF on the 4th, IL8 on the 9th and PDGF on the 18th day of differentiation (Kwak et al., 2013).

The population of glioma CSCs shares a number of intrinsic similarities with NSCs, which includes expression of stem cell markers such as CD133, self-renewal ability and the generation of multi-lineage progeny. In terms of common molecular signatures, both NSCs and glioma CSCs exhibit Y-box binding protein 1 (YB-1), Sox-2, Nestin and Msi1 expression which are lost upon differentiation (Fotovati et al., 2011; Chao et al., 2017). Additionally, glioma CSCs are able to give rise to different neuronal and glial-like cells (Natsume et al., 2013; Bulstrode et al., 2017), which highlights a possible mechanism of glioma CSCs retaining stem cell properties by undergoing asymmetric division. Indeed, the capability to divide asymmetrically with uneven distribution of CSC markers (such as CD133) was reported for glioma cells with stem cell properties (Lathia et al., 2011b), suggesting that asymmetric division may play an important role in tumor maintenance. Moreover, glioma stem cell division showed asymmetry in the distribution pattern of other key stem cell markers, such as Numb, EGFR and Nestin (Cusulin et al., 2015). Numb is a main controller of asymmetric cell division and has been found to be asymmetrically segregated between daughter cells of dividing glioblastoma CSCs, where it colocalized with prominin 1 (CD133; Jiang et al., 2012). Additionally, the asymmetric distribution of the main astrocytic intermediate filament, GFAP, was shown for a small fraction of glioma CSCs (Guichet et al., 2016). Thus, asymmetric division can be a mechanism by which glioma CSCs preserve stem properties whilst also generating the whole repertoire of heterogenic tumor cells within the bulk tumor.

However, other studies have accumulated evidence supporting an alternative theory, postulating that CSCs arise from the inability of transformed NSCs to undergo asymmetric division, leading to continuous symmetric mitosis. As an example, the loss of p53 in cancer was shown to favor symmetrical cell division, while the restoration of p53 was correlated with rescued asymmetric cell division (Cicalese et al., 2009). Therefore, it is also possible that an aberrant increase in symmetrical cell divisions in NSCs contributes to normal cells acquiring a CSC phenotype. Another possibility is that driver mutations occur in NSCs but the actual malignant transformation afflicts their derivative oligodendrocyte progenitor cells or astrocyte precursors. The mechanisms by which p53 mutations affect asymmetry in NSC division are not fully understood, but it would be of interest to investigate if there is a lineage or maturity-specific impact of p53 mutations on the cell differentiation in the brain. Despite p53 mutation not being solely sufficient for astrocytoma formation, it causes an increase in proliferation rate (Gil-Perotin et al., 2006). Additionally, dominant negative p53 can force quiescent stem cells to proliferate and but do not lead to cell differentiation (Ehtesham and Khan, 2015), and in combination with IDH R132H substitution leads to cancer transformation reminiscent of low-grade gliomas (IDH1-mutant TP53 lost astrocytomas; Modrek et al., 2017). Upon introduction of TP53 and NF1 mutations into adult OPCs, they become reactivated and reach the proliferative rate of perinatal OPCs. Interestingly, mutant OPCs firstly turned to a dormant state, until a few cells escaped quiescence and only then malignant transformation ensued (Galvao et al., 2014). This could reflect the physiological process occurring during glioma establishment, when normal slow-cycling stem cells transform into a malignant state, but remains dormant until a specific signal from the surrounding milieu awakes them.

In favor of the symmetrical hypothesis of arising glioma CSCs, it was shown that decreased asymmetry in distribution of neural/glial antigen 2 (NG2), which is a proteoglycan required for normal OPC division, correlates with premalignant, abnormally increased self-renewal and with tumor-initiating potential in the OPC progeny. NG2 is needed for asymmetric segregation of EGFR+ cells to the NG2+ progeny, which is conferred with increased EGF-dependent proliferation and self-renewal, whereas the NG2− progeny differentiate (Sugiarto et al., 2011). Wnt signaling was also proved to contribute to asymmetric brain cell mitosis. Wnt, through the actions of β-catenin, directed asymmetric inheritance of centrosomes and promoted expression of pluripotency genes in β-catenin+ daughter cells (Habib et al., 2013). However, GBM CSCs treated with Wnt activators exhibited decreased proliferation ability and were committed to neuronal differentiation (Rampazzo et al., 2013). Additionally, single-cell RNA sequencing of IDH1-mt gliomas revealed the presence of a normal differentiation program in glioma CSCs across two lineages: astrocytes and oligodendrocytes. It also identified a small population of highly cycling cells with expressed stem cell genes, suggesting that low grade gliomas contain conventional hierarchy with a small population of tumor amplifying cells dividing in normal symmetric fashion (Tirosh et al., 2016).

Thus, glioma CSCs have an additional way of preserving the quiescent cell population, i.e., by asymmetric division allowing preservation of stem cell properties and genetic stability over time. This asymmetry in glioma CSCs is ensured by different distribution of the GFR (EGFR) molecules, transcription factors (e.g., Oct4, Sox2, etc.), membrane glycoproteins and proteoglycans (CD133, NG2) regulating stem cell properties and suppressing differentiation. Markedly, several mutations (p53, NF1) can favor symmetric cell division, which allows to put forward an alternative explanation for the gliomagenesis, i.e., via disrupting the normal asymmetric division of NSCs. The pathways influencing symmetric and asymmetric division of stem cells are summarized in Figure 2C. All in all, the evident contribution of both symmetric and asymmetric cell division plays a role in the aforementioned processes, and highlights the need for a better understanding of the fine molecular mechanisms behind either preservation or the initiation of the CSC quiescence. It also warrants the fine tuning of *in vitro* cell based models available to both basic researchers and clinicians to explore this phenomenon (Mikhailova et al., 2018).

## Glioma CSC Niche

Another mechanism which can support glioma CSC quiescence is associated with niche factors. “Niche” refers to the microenvironment within the specific anatomic location where stem cells interact with other cells or ECM, providing them with stimuli that restrains them from maturation and sustains replication ability. Niche residence can also protect stem cells from aqcuiring mutational errors (Schofield, 1978).

### Glioma Associated Cells

There is no consesus about niche cells or ECM components contributing to microenvironment of stem cells. Recently, it was shown that stem cell progeny provide feedback signals for quiescence or symmetric/assymetric division to stem cell therefore acting as niche cells (Redondo et al., 2017). This feedback includes stimuli to maintain quiescence and preclude excessive or unneeded activation, thus preserving a functional pool of stem cells. Additionally, matured astrocytes were shown to facilitate NSCs proliferation via Wnt7a by activating β-catenin–cyclin D1 pathway or can promote differentiation via β-catenin–neurogenin 2 pathway (Qu et al., 2013). Moreover, it was shown that removal of terminally differentiated cells from the stem cell niche massively activates quiescent stem cells (Hsu et al., 2011). Niche cells surround the stem cell and collect signals provided by the tissue or more distant sites while maintaining the local microenvironment. Long lasting signals or signals with sufficient threshold will be firstly received by the cells in the local milieu which will in turn regulate stem cell behavior (So and Cheung, 2018). In support of this theory, recently, it has been revealed that molecules produced at the site of injuries do not reach stem cells directly, but are translated into stimuli that reprogram niche cells and ECM surrounding stem cells, thus priming quiescent stem cells for activation (Rodgers et al., 2014).

**Figure 3:**
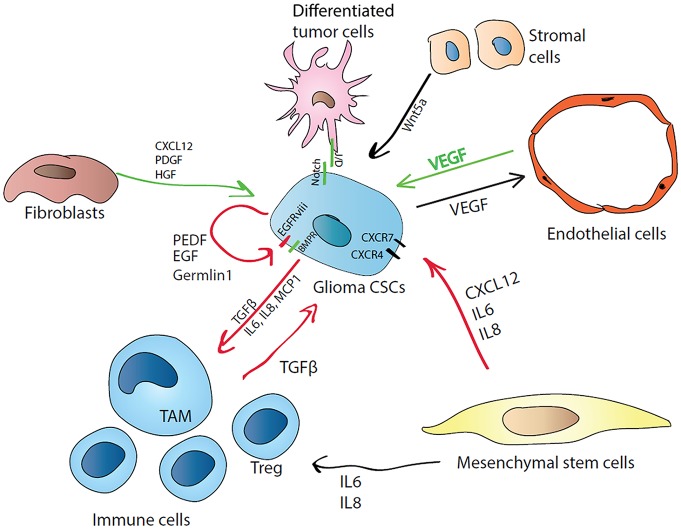
Cellular and molecular regulators of glioma CSC quiescence in the niche. CSCs can regulate their own state by autocrine mechanism by producing differentiation antagonists (Germlin1) or poise themselves to active growth by synthesizing PEDF and EGF, or by expressing constitutively active form of EGFR (EGFRviii). CSCs can attract immune cells by releasing proinflammatory cytokines (MCP1 for monocytes, IL6 and IL8) and induce their immunosuppressive phenotype (M1 macrophages transform into tumor-associated macrophages (TAMs), effector T cells are induced to T regulatory cells (T regs)) by producing TGFβ. In turn, induced tumor-associated immune cells favor the CSC state by producing TGFβ. Mesenchymal cells CXCL12 can activate PI3K/Akt, IP3 and mitogen activated protein kinase (MAPK) pathways via CXCR4 and PLC/MAPK pathway via CXCR7. IL-8 supports glioma CSCs growth and migration towards endothelial cells. CSCs also regulate their nutrition supply by inducing neo-vascularization via vascular endothelial growth factor (VEGF) production. Stromal cells producing Wnt5a can induce CSC quiescence via Ryk receptor. Differentiated tumor cells can regulate CSCs state via cell-cell contact through Notch signaling.

However, tumors are composed of cells that do not respond to external proliferative or static stimulation and are able to evade growth suppression and death signals from the local microenvironment. Another characteristic feature is that cancer cells modulate neighboring cells by producing cytokines or expressing immunosuppressive ligands (Hanahan and Weinberg, 2011). The glioma CSC microenvironment mainly consists of differentiated tumor cells, endothelial cells, pericytes, fibroblasts, normal glia, neurons and immune cells (see Figure 3). The cytoarchitecture of glioma consists of peripheral normoxic tumor cells and hypoxic tumor cells residing in the center as well as necrotic cells in the inner cores (Lathia et al., 2011a). This niche cells maintain glioma CSCs in a dormant state preserving their potential to proliferate and differentiate, at the same time protecting them from chemo- and radiotherapy (Sanai et al., 2005). Glioma CSCs are thought to localize in close proximity to perivascular niches (Flavahan et al., 2013). Glioma CSCs may produce VEGF, promoting vessel growth, while endothelial cells cocultured with glioma CSCs, facilitated the CSC-like phenotype of glioma cells by increasing the expression of Sox2, Olig2 and Bmi1 (Fidoamore et al., 2016). Moreover, endothelial cells may maintain glioma CSC properties by activating the Hedgehog signaling pathway (Yan G. N. et al., 2014). Interestingly, the glioma CSCs may contribute directly to tumor neovascularization, because they can transdifferentiate into endothelial cells (Ricci-Vitiani et al., 2010). Furthermore, glioma CSCs are capable of generating pericytes, indicating an active role for CSCs in the building of an appropriate niche supplied with additional vasculature and nutrients (Cheng et al., 2013).

Additionally, CSCs and endothelial cells combined within the tumor microenvironment can transform normal fibroblasts into cancer-associated fibroblasts (CAFs) possessing increased proliferation and secreting unique cytokines, such as CXCL12, VEGF, PDGF and HGF (Junttila and de Sauvage, 2013). CAFs cocultured with glioma CSCs were shown to serve as feeder cells to supply stemness factors, while their removal led to decreased expression of stem cell markers such as Oct4 and Nanog followed by partial CSC differentiation (Chen et al., 2014). Reciprocally, inhibition of Notch signaling leads to detachment of glioma CSCs from their vascular niche, resulting in increased efficacy of radiotherapy (Hovinga et al., 2010). However, PEDF may also regulate Notch to contribute to stemness in the vascular niche (Andreu-Agullo et al., 2009). Glioma CSCs can produce PEDF and engage in their own autocrine regulation of self-stemness/-renewal, which occurs by activation of EGFRvIII/STAT3/PEDF or Notch/Sox2 signaling.

Mesenchymal stem cells were also reported to contribute to multiple mechanisms favoring cancer cell proliferation, including fostering vasculogenesis and inducing local immunosuppressiveness (Nishimura et al., 2012). These cells can secrete a variety of cytokines promoting cancer stemness through NF-κB pathway, such as CXCL12, IL6 and IL8 (Cabarcas et al., 2011). CXCL12 controls normal stem cell homing in the brain and maintains stem cells in their niche (Maksym et al., 2009). However, in glioma CXCL12 can activate PI3K/Akt, IP3 and MAPK pathways via CXCR4 (Boldajipour et al., 2008) and PLC/MAPK pathway via CXCR7 resulting in increased cell survival in the quiescent state. Interestingly, CXCR4/CXCL12 signaling is remarkably active in the areas surrounding necrotic foci, which contain increased number of quiescent CSCs (Zagzag et al., 2008). IL8 effects are exerted via binding to CXCR1 and CXCR2, which mediates the invasion of glioma cells (Zhu et al., 2012). Recent findings describe IL-8 as a critical mediator in supporting glioma CSC growth and migration towards endothelial cells, which is one of the possible mechanisms forcing their perivascular colocalization within the tumor niche (Infanger et al., 2013).

Additionally, mesenchymal stem cells attracted to the glioma CSC niche can stimulate tumor progression by producing the BMP antagonist, Gremlin 1, which antagonizes differentiation signals through the inhibition of p21WAF1/CIP1, which is a key CSC signaling node. BMP family members stimulate NSCs to differentiate to astrocytes, and therefore, they were proposed as candidates for application in anti-CSC therapies, as per they could assist CSCs differentiation (Carén et al., 2016), but, paradoxically, gliomas express high levels of BMPs. However, it was further demonstrated that glioma CSCs highly express Gremlin1 as their protection from endogenous BMP (Yan K. et al., 2014).

Glioma CSCs are capable of recruiting numerous cell types by secreting chemokines. Cytokines attract and induce tumor-associated macrophages (TAMs), tumor-associated neutrophils and other suppressor cells. Attracted macrophages secrete TGF-β and recruit T regulatory cells (Tregs) that ensure maintenance of immunosuppression conditions (Chanmee et al., 2014). Induced immunosuppressive cells protect the tumor by producing IL6, TGF-β and TNFα, upregulating the NF-κB molecular pathway which in turn activates Snail, Slug and Twist to stimulate self-renewal (Kitamura et al., 2015).

In normal stem cell niches, tight attachment of stem cells to the niche through cell-cell contacts is crucial for preventing their differentiation and for localizing factors that maintain self-renewal physically adjacent to niche (Borovski et al., 2011). CSCs also anchor to their niche cells to preserve stemness, as direct cell contact is particularly necessary for Hedgehog and Notch pathways. However, Notch signaling was shown to inhibit brain tumor initiation and growth, although, it is possible that transformed glioma cells were turned to a quiescent state (Giachino et al., 2015). Meanwhile, normal glial cells may also be utilized for cell-to-cell contacts to tether glioma cells (Riquelme et al., 2008).

### Glioma Associated Extracellular Matrix

Apart from neo-vascularization and the effects of immune and stromal cells within the tumor microenvironment, an important role is played by the ECM, which confers the structural scaffold. It contains fibrous proteins such as elastin, collagens, fibronectin, laminins, as well as cellular proteases, such as cathepsins, matrix metalloproteinases and kallikreins, as well as globular proteins including integrins (Melzer et al., 2016). Laminin from tumor cells can alter glioma CSC phenotype derived from the perivascular niche (Lathia et al., 2012). Moreover, laminin-111 was shown to induce quiescence of breast epithelial cells in a three-dimensional cell culture system by depleting nuclear-associated actin (Spencer et al., 2011).

Still it is evident that the tumor milieu provides supporting growth signals, protection from active immune cells and contributes to therapy resistance. However, the nature of CSC regulation by its tumor niche remains unresolved. An intriguing question is whether the CSC fate is regulated by intrinsic (autocrine or autonomous) stimuli or through interactions with the local environment. Another open question is if CSCs within their local niche are confined to a closed domain that is not receiving systemic stimuli.

## Concluding Remarks and Future Directions

Taken together, in healthy conditions a quiescent state protects normal stem cells from senescence and exhaustion thus preserving the multipotent ability to reconstitute brain components. Recent findings support the hypothesis that all remote stimuli affect stem cells not by direct stimulation but rather by changing the niche state which in turn regulates resident stem cells, thus protecting them from unnecessary activation. In a disease state, glioma CSCs can also preserve stem cell properties by actively changing the oxygen supply, nutrient availability and regulating the state of different neighboring cells. Glioma CSCs have several major molecular mechanisms implicated in maintaining their active quiescent state. Those mechanisms include the aberrant expression of the genes facilitating key regulatory processes. Such processes include altered cellular growth and survival, which commonly happen due the acquisition of the mutations leading to either constitutive EGFR activation (EGFRvIII), or GOF IDH1/2 and p53 mutations, globally affecting gene expression and other chromatin-dependent processes. Additionally, glioma CSCs can preserve their stem cell properties by their ability to asymmetrically divide which allows simultaneous retention of replication ability and genome stability while propagating the tumor mass. This point also favors the theory that high-grade gliomas most probably originate from NSCs in the brain SVZ. In addition, glioma CSCs actively change their microenvironment, creating a comfortable cancer niche by attracting endothelial cells for vessel construction and inducing immune cells to the immunosuppressive state. Thus, glioma CSCs possess a wide range of survival mechanisms to evade targeted therapy as blockage of one specific pathway will be compensated by a range of other overlaying molecular cascades. The promising option to overcome this problem is differentiation therapy which may force glioma CSCs to purposefully re-enter the cell cycle in order to be eliminated by chemotherapy when they are no longer protected by a dormant state. Unfortunately this option has certain disadvantages, as induction of tumor cell proliferation is not an ideal treatment, however, in the case of high-grade cancers, treatment almost always implies the hard choice between possible complications and a possible benefit from treatment. Thus, future perspectives include therapeutic manipulations of the stem cell niche to induce differentiation of glioma CSCs as a novel approach of anticancer therapy.

Another possibility is to carefully assess disease development in each patient and reveal the dominant cancer maintaining mechanism by considering the particular glioma molecular profile (genetic alterations/aberrant gene expression), thus bringing glioma treatment to the era of precision medicine. In this case, some patients can be treated with immunotherapy, interfering with several important pathways by treatments such as TGFb inhibitors and agents blocking secretion of anti-inflammatory factors. It is also possible to push CSC differentiation by blocking BMP and EGFR mediated signals, to prevent tumor vascularization by inhibiting VEGF, to increase tumor sensitivity by interfering with niche adhesion molecules (N-Cadherin and VCAM1). In addition, finding the markers for quiescent CSCs may contribute to their specific eradication by targeted therapy. Further understanding the CSC developmental hierarchy and mechanism of choice between quiescent or proliferative state will allow new approaches to glioma therapy.

## Author Contributions

VG, SR, AK, EC and AR wrote the manuscript. VK and NS drew figures and wrote legends to them. All authors approved the final manuscript.

## Conflict of Interest Statement

The authors declare that the research was conducted in the absence of any commercial or financial relationships that could be construed as a potential conflict of interest.
